# Extracellular vesicle-induced cyclic AMP signaling

**DOI:** 10.1016/j.cellsig.2022.110348

**Published:** 2022-04-30

**Authors:** Aritra Bhadra, April K. Scruggs, Silas J. Leavesley, Naga Annamdevula, April H. George, Andrea L. Britain, Christopher M. Francis, Jennifer M. Knighten, Thomas C. Rich, Natalie N. Bauer

**Affiliations:** a Department of Pharmacology, College of Medicine, University of South Alabama, Mobile, AL, United States of America; b Department of Chemical and Biomolecular Engineering, College of Engineering, University of South Alabama, Mobile, AL, United States of America; c Department of Physiology and Cell Biology, University of South Alabama, Mobile, AL, United States of America; d Center for Lung Biology, College of Medicine, University of South Alabama, Mobile, Alabama

**Keywords:** Extracellular vesicles, Cyclic adenosine monophosphate, Second messenger, Microvesicle

## Abstract

Second messenger signaling is required for cellular processes. We previously reported that extracellular vesicles (EVs) from stimulated cultured endothelial cells contain the biochemical second messenger, cAMP. In the current study, we sought to determine whether cAMP-enriched EVs induce second messenger signaling pathways in naïve recipient cells. Our results indicate that cAMP-enriched EVs increase cAMP content sufficient to stimulate PKA activity. The implications of our work are that EVs represent a novel intercellular mechanism for second messenger, specifically cAMP, signaling.

## 1. Introduction

Extracellular vesicles are released from parent cells under stimulated conditions, such as inflammatory mediators during disease. The number of EVs released and the content of the EVs can be altered dependent on stimulus and their function can be either harmful or beneficial such as in the case of reparative EVs from mesenchymal stem cells [1–6]. EVs are intact vesicles under 1 μm in diameter. They can be collected from serum, plasma, urine, and cell culture media via ultracentrifugation [7]. The term extracellular vesicle currently comprises a heterogeneous population of circulating, intact, membranous vesicles including exosomes and microvesicles [8–10]. While early data in the field suggested each type of these intact vesicles possess characteristic features, more recent work reveals that the vast majority of distinguishing features are better described as distributions that overlap among types of vesicles [11]. Thus, the term extracellular vesicle is used here to describe the mixed population of vesicles.

Signaling conferred by EVs includes delivery of functional proteins as well as RNAs and miRNAs that influence recipient cell behavior [5,12,13]. For example, transfer of the chemokine receptor CCR5 by EVs to CCR5-null cells confers functional receptor expression and allows for HIV-1 infection [14]. Tumor cell-derived EVs can deliver active EGFRvIIIs that promote activation of EGFRvIII-related genes to recipient cells, suggesting that incorporation of the EV-delivered receptors can induce altered proliferation [15]. In a series of studies to examine miRNA delivery, Cy3-labeled miR-92a generated in leukemia cells and packaged in EVs was delivered to human umbilical vein endothelial cells. The EV-delivered miRNA reduced integrin α-5 expression indicating that EV delivered miRNAs functioned as expected in parent cells [16]. Further studies have elucidated a number of extracellular vesicle-associated or extracellular vesicle-delivered miRNAs including miR-145, miR-9 and miR-223 [17–20]. While our understanding of extracellular vesicle-mediated signaling is expanding rapidly, there are several gaps that remain, including whether EVs alter second messenger signaling pathways.

To address this knowledge gap, we sought to determine whether EVs contain the canonical second messenger cAMP and recently published that EVs isolated from pharmacologically stimulated endothelial cells do contain cAMP [21]. In the current study we hypothesized that treatment of naïve endothelial cells with these cAMP-enriched EVs would induce increased cellular cAMP. To address the question of whether EVs contain sufficient cAMP to induce these responses, we developed mathematical models of the cAMP signaling pathway in endothelial cells. Simulations of this model indicated that EVs contain sufficient cAMP to trigger marked increases in PKA activity in target cells. We then experimentally confirmed that our cAMP-enriched EVs are sufficient to induce phosphorylation of downstream effectors of PKA. Our data suggest that following treatment of naïve cells with cAMP-enriched EVs, cAMP content is increased, PKA activity increased, and these events trigger phosphorylation of both endogenous and exogenous targets. Taken together, these observations suggest that EVs can indeed induce cAMP-mediated responses in endothelial cells.

## 2. Materials and methods

### 2.1. Cell culture and extracellular vesicle collection

Rat pulmonary microvascular endothelial cells (PMVECs) were obtained from the Center for Lung Biology cell culture core and maintained as previously described [22]. Briefly, cells were grown in Dulbecco’s modified Eagle medium (DMEM) with 10% heat-inactivated fetal bovine serum (Invitrogen #10082, Carlsbad, CA) and 1% penicillin/streptomycin (Invitrogen #15140) at 37 °C, in 21% O_2_ and 5% CO_2_. EVs were collected from six 100 mm culture dishes at near confluence. Culture media was removed, cells were washed with PBS, and serum-free media was added to cells for the duration of treatment. PMVECs were treated with rolipram (10 μM, 5 min, Alfa Aesar #J67177, Ward Hill, MA), isoproterenol (1 μM, 10 min, EMD Millipore Corp. #420355, Billerica, MA), both, or a vehicle control (DMSO) [21]. After treatment, the media was removed, pooled, centrifuged for 10 min at 1000 × G to remove cell debris, and ultracentrifuged for 1 h at 100,000 × G at 4 °C to pellet EVs per our previously reported protocol [21,23,24]. Vesicles were resuspended in sterile PBS and kept at 37 °C before addition to cells.

### 2.2. EV transmission electron microscopy (TEM) and nanoparticle tracking analysis (NTA)

After collection, EVs were fixed using 2% paraformaldehyde and deposited on carbon-coated EM grids and incubated for 20 min for adsorption. Following incubation, the grids were incubated with 1% glutaraldehyde for 5 min and followed by 8 washes with ultra-pure water. The samples were then counterstained with double stain uranyl/lead citrate reagent. A Phillips CM100 TEM was used to image the samples.

The particle size distribution of isolated extracellular vesicles was analyzed using ZetaView (Particle Matrix, Germany). Following isolation from media, EVs were resuspended in 2 mL ultrapure water and analyzed at room temperature using the following instrument settings: maximal area 1000, minimal area 8 and minimum brightness 25. The data were acquired in a two-cycle measurement over 11 positions and were analyzed using the software ZetaView version 8.05.10. For both control and stimulated conditions this is 2.6 × 10^9^ EVs. This total number of EVs, 2.6 × 10^9^ EVs from 6 × 100 mm dishes of cells, were used in every experiment.

### 2.3. Transfection of FRET sensors

PMVECs were seeded on laminin-coated 24 mm glass coverslips in 6 well dishes and grown to 70% confluence before transfection. Cells were transfected using lipofectamine 3000 transfection reagent (Thermo Fisher #L3000015) with 2.5 μg of plasmid encoding the H188 FRET-based cAMP reporter per well [25]. For some experiments, cells were transfected with the donor or acceptor alone to obtain their spectral signatures or to perform photobleaching controls. Photobleaching and vehicle controls were performed using the same laser settings across all experiments. Cells were assayed 48 h after transfection. Three to five cells per field were analyzed and single cells with intensity peaks greater than the background fluorescence (at 505 nm) were selected for post-acquisition analysis.

### 2.4. Hyperspectral imaging and analysis

Microscopy was performed using a Nikon A1R inverted confocal microscope equipped with 60× water immersion objective (Plan Apo VC 60× DIC N2 WI NA-1.2). A confocal pinhole radius of 84.3 μm was used. Hyperspectral image stacks were acquired at each time point with an effective optical section thickness of 1.73 μm. Excitation wavelengths were 405.7 and 562.9 nm. Emission wavelengths were acquired from 414 to 724 nm in 10 nm increments (31 wavelength bands; the 564 nm wavelength band was disabled to avoid artifacts due to the mechanical finger automatically blocking this detector when the 561 nm laser is used).

Coverslips with transfected cells were placed in a humidified microscope stage for imaging and experiments were performed at room temperature in 1 mL PBS. In our experience experiments performed at room temperature are less susceptible to photobleaching than experiments performed at higher temperatures. Cells were labeled with Draq5 (25 μM, BioStatus Limited #DR51000, Shepshed, UK) for 10 min in order to identify nuclei. Baseline fluorescence signals were acquired for 90 s prior to addition of EVs. Spectral stacks were acquired every 30 s for 15 min. Forskolin (50 μM, EMD Chemicals #344270, San Diego, CA), an activator of adenylyl cyclase, was added to cells at 17 min as to confirm the responsiveness of the FRET-based cAMP probe.

Custom hyperspectral image analysis and linear unmixing scripts were developed in MATLAB identify fluorescence spectra and to subsequently estimate FRET efficiency, as described previously [26,27]. In brief, the distributions of each fluorophore in the spectral library (Turquoise, Venus, Draq5, and background) were unmixed from spectral image stacks based upon the least squared error criteria. Unmixed Turquoise and Venus images were used to calculate FRET efficiency. Unmixed images were used to identify cell cytosol based upon threshold masks for the nucleus and background. The nucleus and background regions were excluded from FRET calculation. FRET efficiency was then mapped to cAMP concentration. cAMP image data were processed using adaptive thresholding and region of interest tracking software developed in house. Images depicting cAMP concentration were filtered temporally using Savitzky-Golay filtering and adaptive thresholding approaches (OTSU) were applied to generate masked images indicating cAMP signals above the threshold. The K_D_ of cAMP sensors, including the H188 sensor used in this study, have been difficult to measure in intact cells. Therefore, cAMP levels were plotted as a function of the K_D_ of the sensor. Quantified data are averaged cAMP levels in a single confocal slice at the specificized timepoint.

### 2.5. Collection of cell lysates for Western blot

PMVECs were grown to approximately 70% confluence in a 6-well plate (3 mL per well). Isolated I/R EVs (2.6 × 10^9^ EVs per well) were resuspended in EV-free media, added to two wells and incubated for 10 min at 37 °C. Two wells were treated with 10 μM rolipram and incubated at 37 °C for 5 min, then treated with 1 μM isoproterenol and incubated at 37 °C for 10 min. Two wells were treated with vehicle and incubated at 37 °C for 5 min, then treated with serum free media and incubated at 37 °C for 10 min. Cells from each of the 6 wells were lysed on ice in RIPA buffer containing 1% protease inhibitor (Sigma, P1860) and 1% phosphatase inhibitor (Sigma P0044). Cells were scraped with a cell scraper and aspirated into a syringe with a 25G needle, then transferred to microfuge tubes. Cell lysates were centrifuged at 10,000 ×*g* for 10 min at 4 °C and pellet was discarded.

PMVECs were pretreated with 25 μM propranolol (a non-selective β-blocker) for 1 h to inhibit β-receptors. Following pretreatment, PMVECs were treated with either isoproterenol or I/R stimulated EVs as above for 10 min. Whole cell lysates were collected and analyzed for VASP/p-VASP and PKA activity as described below.

### 2.6. Mathematical modeling

Differential equations describing basal cAMP production, degradation, and diffusion of cAMP were used to model baseline cAMP distributions in the target cell. Delivery of EV contents was modeled as the instantaneous addition of cAMP to the target cell. The model was based upon previous two-compartment models that included simplified descriptions of the spatial spread of cAMP signals, in which cAMP levels in the near plasma membrane compartment readily equilibrate, but the flux between the near-membrane compartment (C1) and the bulk cytosol (C2) is markedly hindered. [28,29] Rates of cAMP turnover and diffusion between compartments were based upon parameters used in other studies [28–30]. Mathematical descriptions of EV-target cell interactions were coded in the MATLAB programming environment using the ODE23s solver. Initial conditions were obtained by running simulations to equilibrium with basal adenylyl cyclase and phosphodiesterase activities; basal cAMP is approximately 0.1 μM in C1 and 0.05 μM in C2. Release of cAMP from a single EV in either the near-membrane compartment (C1) or the cytosolic compartment (C2) of target cells was assumed to occur instantaneously. Equations describing cAMP concentration are below.

(1) $$\frac{d[cAMP{]}_{C1}}{dt}=\frac{AC_{basal.c1}-E_{PDE-C1}-k_{1}*[cAMP{]}_{C1}+k_{2}*[cAMP{]}_{C2}}{V_{1}}$$

(2) $$\frac{d[cAMP{]}_{C2}}{dt}=\frac{AC_{basal.C2}-E_{PDE-C2}-k_{2}{\;}^{*}[cAMP{]}_{C2}+k_{21}{\;}^{*}[cAMP{]}_{C1}}{V_{2}}$$

(3) $$E_{PDE-C1}=\frac{V_{max1}{\;}^{*}[cAMP{]}_{C1}}{[cAMP{]}_{C1}+K_{m1}{\;}^{*}\left(1+\frac{K_{I}}{[I}\right)}$$

(4) $$E_{PDE-C2}=\frac{V_{max2}{\;}^{*}[cAMP{]}_{C2}}{[cAMP{]}_{C2}+K_{m2}{\;}^{*}\left(1+\frac{K_{I}}{\left[I\right]}\right)}$$

where C1 and C2 represent compartments 1 and 2, and $E_{PDE-C1}$ and $E_{PDE-C2}$ represent PDE activity in C1 and C2. Additional parameter descriptions and values are provided in Table 1.

### 2.7. Western blot and PKA activity

A Precision Red (Cytoskeleton, Inc. #ADV02, Denver, CO) protein assay was used to determine protein concentration. Cell lysates containing 20 μg of protein were mixed with SDS sample buffer and heated at 95 °C for 10 min. Samples were loaded into a BIS-TRIS gel (NuPAGE Novex 4–12% agarose, Invitrogen # NP0335BOX) and electrophoresed at 120 V for 2 h. Proteins were transferred to a polyvinyl difluoride (PVDF) membrane at 100 V for 45 min on ice. Membranes were washed in PBS and blocked with 5% bovine serum albumin (BSA, Sigma #A7906) in PBS on a rocker at room temperature for one hour.

Antibodies to VASP total protein (CS 3132S, Cell Signaling Technology, Danvers, MA) and VASP phosphorylated at Ser 157 (CS 3111P, PKA specific phosphorylation site, Cell Signaling Technology) were diluted to working concentration (1:1000) in 5% BSA in PBS and placed on a rocker at 4 °C overnight. Membranes were washed in TidyBlot wash buffer (5% milk/PBS with 0.1% Tween-20) according to manufacturer protocol, then Tidy Blot (1:200 concentration in 5%milk/PBS; Bio-Rad Laboratories # STAR209P, Hercules, CA) was added to membranes that were subsequently placed on a rocker for 1 h at room temperature. Subsequent washes were: 4 × 5 min in wash buffer, 3 × 5 min in PBST and 2 × 5 min in PBS. Supersignal West Femto chemiluminescent substrate (Thermo Fisher #34095) was applied to enhance visualization of proteins as membranes were developed. Analysis of PKA activity was also confirmed with the PKA ELISA (Thermofisher) following the same treatment protocol as the Westerns.

### 2.8. Mass spectrometry analysis of EV isoproterenol and rolipram content

For isoproterenol, samples were prepared using a manual isolations procedure on a C18 column. Standards of isoproterenol were used to determine the optimal percentage of organic solvent needed for elution from the C18 column. 250 μL of media was diluted with 750 μL 0.1% trifluoroacetic acid (TFA), and loaded onto a previously washed and equilibrated C18 column. The flow-through was collected (~1 mL), as well as a 2 mL elution with 20% acetonitrile (ACN) collected in a separate tube. Both the flow-through and 20% fractions were brought to dryness in a SpeedVac concentrator. The flow-through was resuspended into 50 L A1 solvent and combined with the 20% fraction for MS analysis. The standard curve was prepared using the same protocol as the samples. All measurements made were within 1.0 ppm accuracy.

Samples were prepared using protein precipitation and HPLC isolation. 200 μL of ice-cold acetone was added to 50 μL of sample, vortexed, and placed at −20 °C for 10 min to promote protein precipitation. The samples were then centrifuged at 16.1 rcf for 10 min at 4 °C. 240 μL supernatant was transferred to an HPLC vial and brought to dryness in a SpeedVac concentrator. Samples were resuspended into 50 μL 50:50 A1: B1 solvents for HPLC isolation. The C18 gradient method was used, with a gradient from 20% to 90% ACN and a flow rate of 100 μL/min. 10 μL of each sample was injected onto an Agilent 300SB-C18 column (2.1 × 150 mm) equipped with a C18 guard column. The second fraction (rolipram) was collected from 25 to 28 min. Fractions were brought to dryness. The samples were resuspended into 20 μL 50:50 A1:B1 for MS analysis. A standard curve was prepared using the same protocol as the samples. Content of individual analytes in samples (relative abundance) are normalized to cell counts.

### 2.9. Statistics

All statistical analyses were performed in GraphPad Prism version 9. Data are presented as the mean ± standard error of the mean. Student’s *t*-test was performed on data sets with two groups and one-way analysis of variance tests (ANOVA) with Tukey’s multiple comparison post tests were performed on data sets with more than two groups. A *P*-value less than 0.05 was considered significant.

## 3. Results

### 3.1. Extracellular vesicle treatment increases cellular cAMP content

**Fig. 1.**
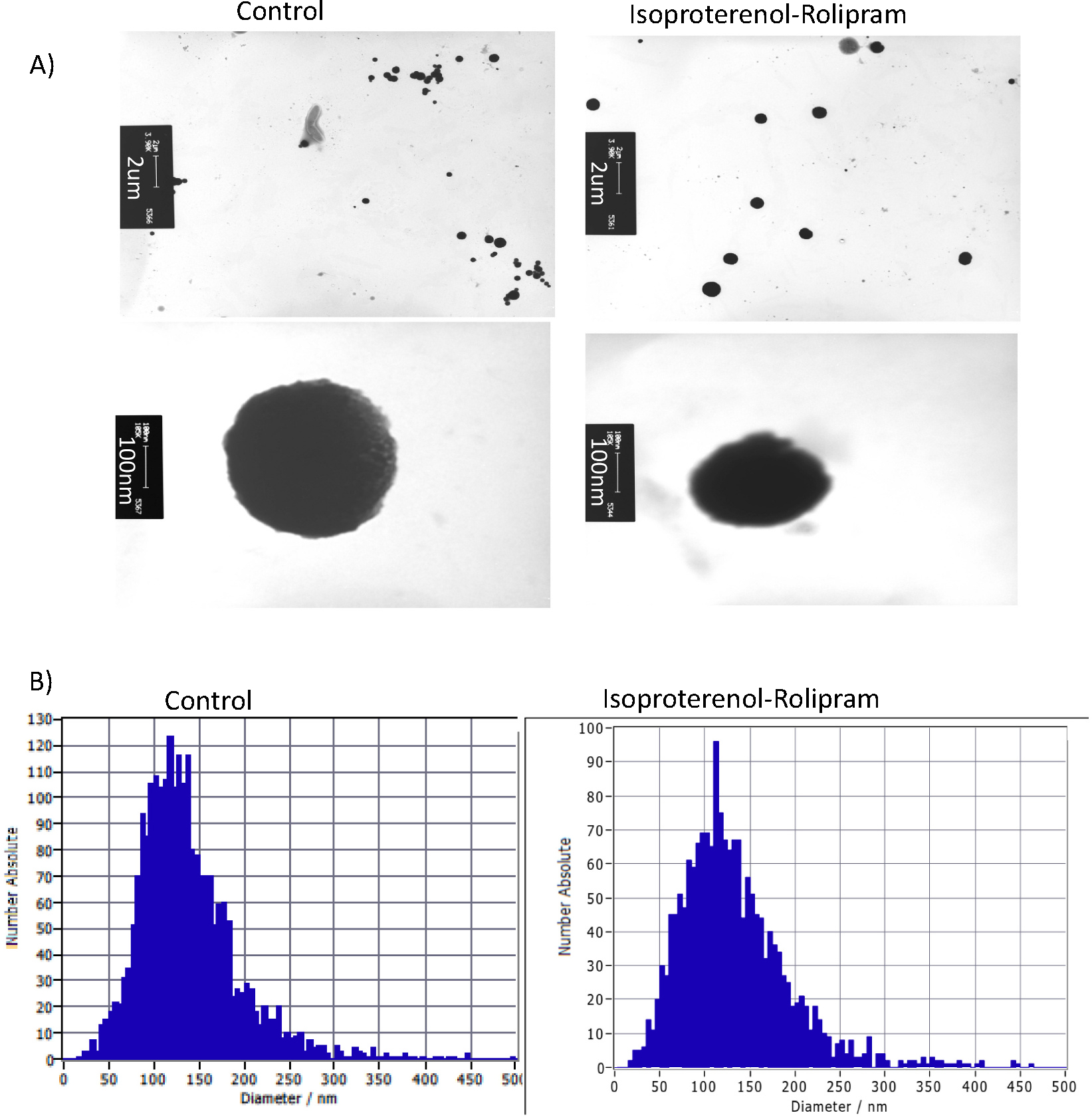
Transmission electron microscopy (TEM) and nanoparticle tracking analysis (NTA) of extracellular vesicle (EVs). (A) EVs were isolated from cell culture medium of control or isoproterenol and rolipram-treated (I/R) cells then fixed and stained for TEM. EVs were intact, dense and heterogenous vesicles. (B) The particle size distribution for extracellular vesicles isolated from control and IR-treated cells. NTA results indicate a range of heterogenous, submicron EVs with a similar median size distribution, *n* = 3.

Our group recently published that pharmacologically stimulated pulmonary microvascular endothelial cells (PMVECs) release EVs containing significant levels of cAMP [21]. These studies were all performed using the β-receptor agonist isoproterenol at a saturating dose to maximally stimulate transmembrane adenylyl cyclase activity, and the phosphodiesterase inhibitor rolipram, to retain maximal cAMP content (I/R). Therefore, our goal with the current manuscript is to recapitulate these conditions to maximize cAMP content of the released EVs, then to determine if these EVs induce signaling events in naïve cells. First, we confirmed that we had intact, identifiable EVs by transmission electron microscopy for visualization and nanoparticle tracking analysis for diameter [10]. These are the two gold standards for acceptable EVs set forth by the International Society of Extracellular Vesicles [10]. Our data indicate that we have intact, circular EVs (Fig. 1A; TEM). Since TEM is not a quantitative measure, but only a snapshot of a sample, we confirmed diameter and number of EVs between isoproterenol/rolipram (I/R) and control cells using nanoparticle tracking analysis (NTA) and illustrate that the EVs are in a similar size range and number between the two conditions (Fig. 1B). We previously reported that the number of EVs does not change with maximal cAMP stimulation, but that the content of cAMP is increased in the EVs and these data confirm this observation [21].

**Fig. 2.**
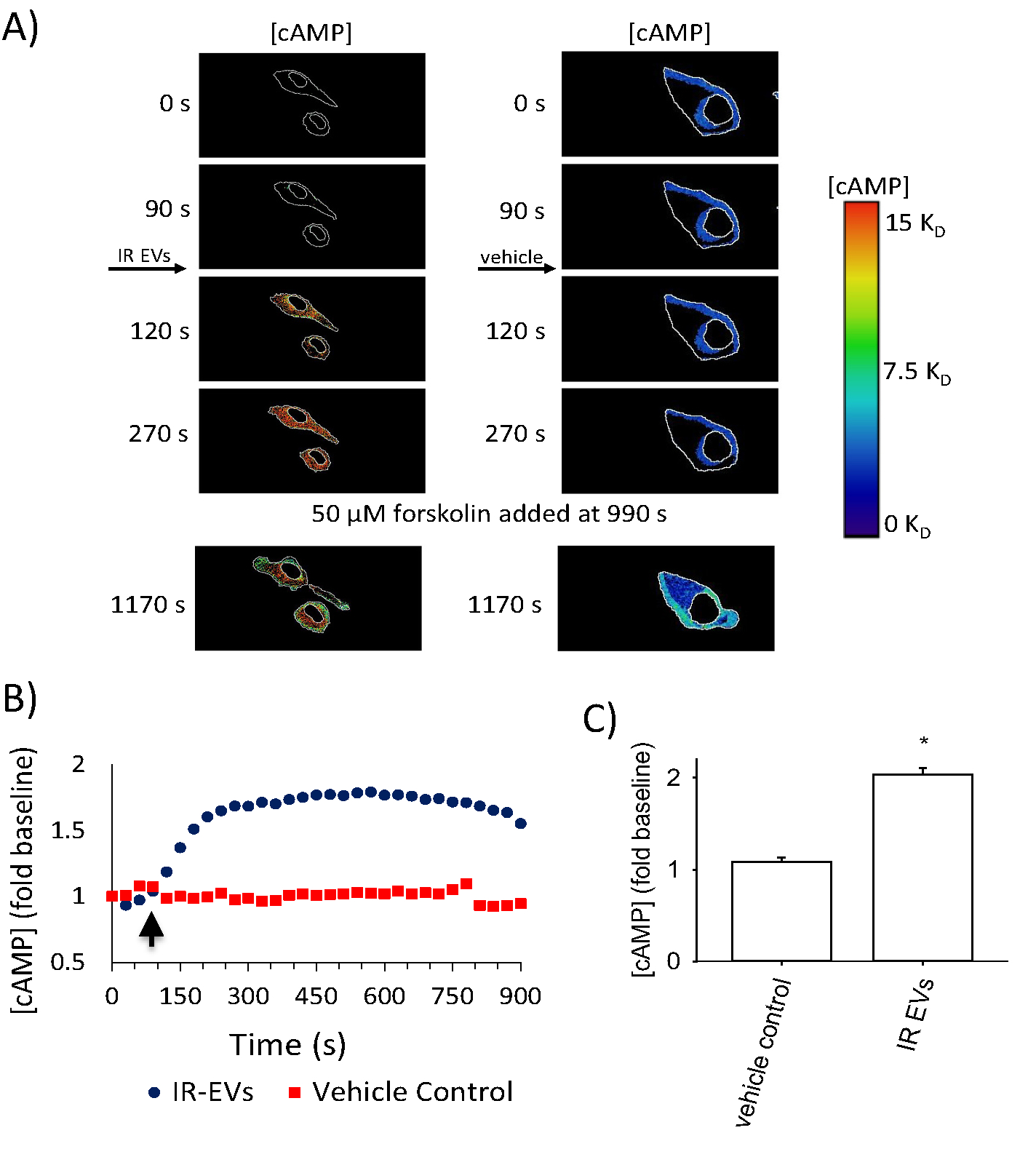
IR EVs trigger increased cAMP levels in PMVECs. IR EVs or vehicle control were added to PMVECs transfected with the H188 cAMP probe. cAMP levels were estimated as described in the Materials and Methods. (A) cAMP levels increased in the cells treated with IR EVs but not in cells treated with vehicle control (left panels). cAMP levels are as indicated by the color bar − hot colors indicate high cAMP and cool colors indicate the low cAMP. Outlines of the cell border (indicated by the white line at the cellular periphery) were obtained using the donor + acceptor signal. (B) A representative time-course of cAMP accumulation triggered by the addition of EVs (blue circles) of vehicle control (red squares) at 90 s (indicated by arrow). Data are representative of 6 experiments. (C) Addition of IR EVs triggered significant increases in intracellular cAMP levels in target PMVECs. Data were measured at the timepoint 5 min after addition of IR EVs. (* *p* < 0.01; *n* = 6). (For interpretation of the references to color in this figure legend, the reader is referred to the web version of this article.)

Since EVs deliver proteins and RNAs to cells, using the PMVECs as a model system, we next sought to determine whether cAMP-containing EVs, when delivered to naïve cells, could increase cellular cAMP and ultimately activate downstream effectors. We utilized the H188 cAMP sensor to measure cellular cAMP in live cells. H188 is designed around a cAMP binding site of Epac sandwiched between a fluorescence donor and acceptor [25]. FRET occurs when the fluorophores are in close proximity and the donor emission spectrum overlaps with the acceptor excitation spectrum [31]. FRET levels are high when cAMP levels are low; as cAMP levels increase the reporter undergoes a conformational change and FRET decreases. Upon addition of IR EVs, cAMP levels increased, reaching a maximum within 5 min (Fig. 2A and B). Our data indicate a 2-fold increase in cAMP levels 5 min following addition of IR EVs (Fig. 2C). These data clearly suggest that IR EVs trigger increases in intracellular cAMP levels in PMVECs.

### 3.2. Mathematical models predict that cAMP-containing EVs are sufficient to activate protein kinase A (PKA) in vesicle treated cells

**Fig. 3.**
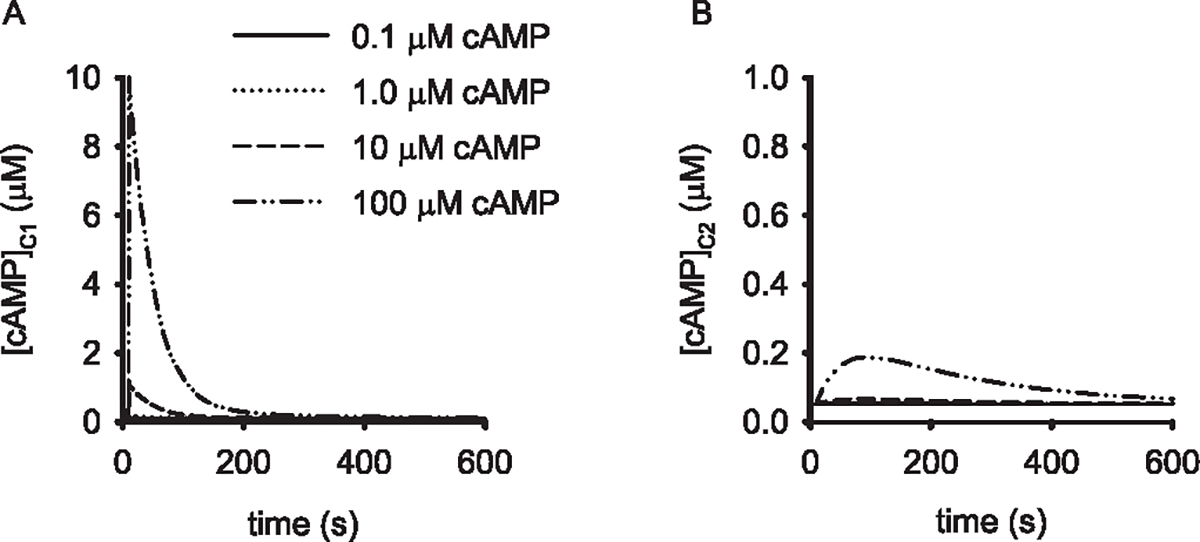
Simulations of EV-mediated cAMP release in the near-membrane compartment of an endothelial cell. (A) cAMP levels in the near-membrane compartment (C1) triggered by release of cAMP from an individual EV into C1 at 10 s. [cAMP] EV cAMP concentrations are as indicated in the legend. (B) cAMP levels in the cytosolic compartment (C2) triggered by release of cAMP from an individual EV into C1. EV cAMP concentrations are as indicated in the legend of panel (A). It is apparent that EVs containing relatively high cAMP concentrations can activate PKA in near-membrane compartments (PKA EC_50_ ≈ 0.1 μM cAMP). It is likely that PKA activity in C1 would remain high enough to induce sustained activation of PKA in C1. However, PKA activation would be lower in amplitude and transient in the remainder of the cell (C2).

Our experimental data suggest that EVs contain cAMP and that EV interactions with cells are sufficient to increase cAMP levels in target cells. However, it was not clear to us whether there was sufficient cAMP contained in a single EV or, potentially, released from several EVs, to trigger sustained increases in PKA activity in target cells. To address this question prior to initiating experiments, we utilized a compartmental model of cAMP signaling as described previously [28–30]. Briefly, the mathematical model includes both a near-membrane compartment (C1) and a cytosolic compartment (C2) (Fig. 3A and B, respectively). The near-membrane compartment was 4% of the total cellular volume. cAMP rapidly diffuses (equilibrates) in each compartment; however, the flux of cAMP between compartments was markedly hindered. This basic model has been used to describe a cAMP signaling in a wide variety of cell types, reviewed in [32].

**Fig. 4.**
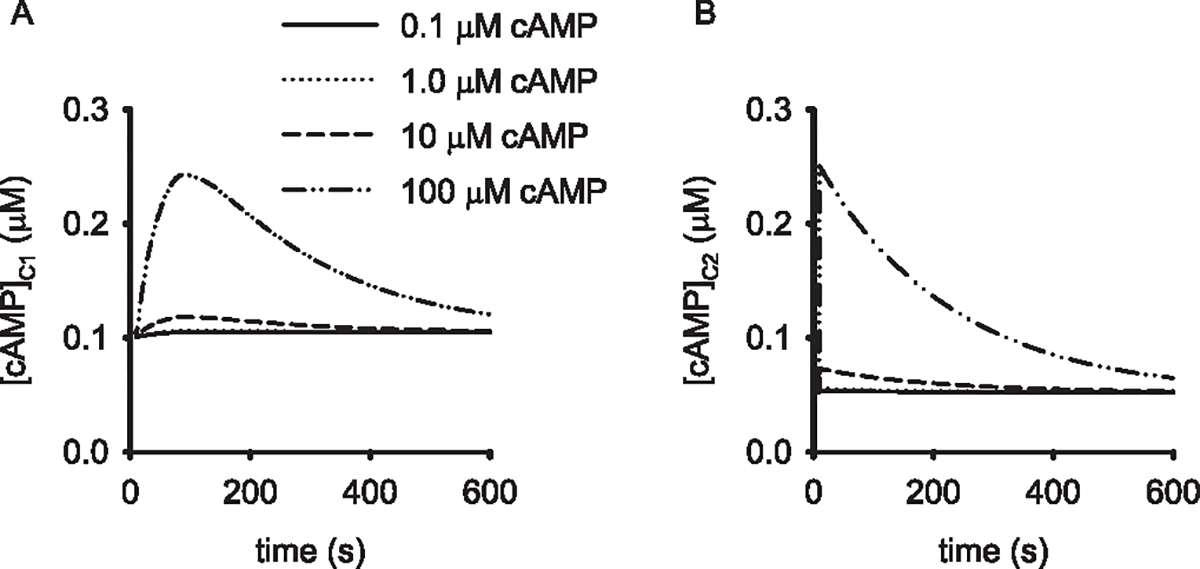
Simulations of EV-mediated cAMP release in the cytosolic compartment of an endothelial cell. (A) cAMP levels in the near-membrane compartment (C1) triggered by release of cAMP from an individual EV into C2 at 10 s. EV cAMP concentrations are as indicated in the legend. (B) cAMP levels in C2 triggered by release of cAMP from an individual EV into C2. EV cAMP concentrations are as indicated in the legend of panel (A). Release of cAMP from an EV with high cAMP concentration into a large cellular volume (e.g. the bulk cytosol depicted by C2) would lead to sustained activation of PKA in C2 for *>*5 min and a transient increase in cAMP in C1.

EV release of cAMP into the target cell was assumed to occur as an instantaneous bolus delivered at 20 s. Release of cAMP from an EV into the near-membrane compartment triggered a rise in cAMP levels with an exponential decay due to both PDE activity and cAMP flux into C2 (Fig. 3). cAMP levels in EVs were modeled as 0.1, 1, 10, or 100 μM, as indicated. These levels of cAMP were chosen to reflect the range agonist-induced free cAMP levels measured in cells and tissues using a variety of biochemical, electrophysiological, and fluorescence/FRET based approaches [33]. Simulations indicated that the release from EVs containing 10 and 100 μM cAMP into the near-membrane compartment (C1) would induce spikes in cAMP in C1 in excess of 1.5 and 9 μM, respectively (Fig. 3A). These cAMP levels would be sufficient to trigger sustained increases in PKA activity in C1 as the EC_50_ of PKA is approximately 0.1–0.2 μM. Increased cAMP levels in C1 would also lead to transient increases in cAMP and PKA activity in C2 (Fig. 3B). In contrast, release of even 100 μM cAMP from an EV directly into C2 was only sufficient to trigger modest, transient increases in cAMP in C1 and C2 (Fig. 4A and B). However, these modest increases in cAMP would still lead to sustained activation of PKA, and thus a shift in relative activities of PKA and phosphatase in both compartments. These simulations suggested that testing of the hypothesis that cAMP-EVs could deliver sufficient cAMP to activate PKA signaling to downstream substrates was feasible and should be tested.

### 3.3. EV treatment results in increased phosphorylation of VASP at the consensus PKA site

Our analysis of changes in global cAMP content with the H188 FRET sensor suggested that total cellular cAMP content was increased in the presence of EVs. Our simulations suggested that this rise in cAMP induced by EVs was sufficient to stimulate PKA activity. We next determined experimentally whether the cAMP increase by EVs was sufficient to induce phosphorylation of a downstream PKA-substrate. We selected vasodilator-stimulated phosphoprotein (VASP) as our downstream target of PKA activation [34–36].

**Fig. 5.**
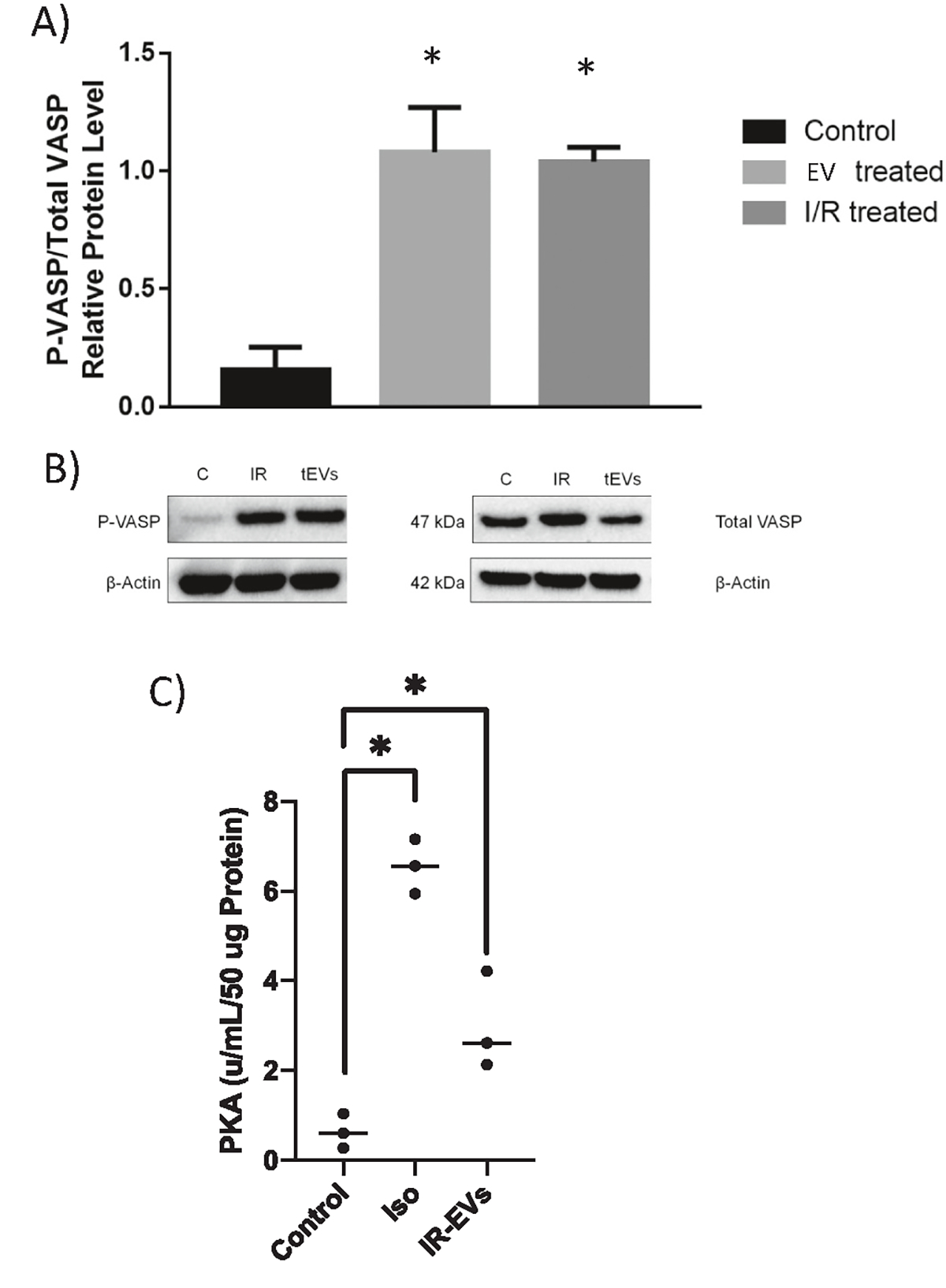
EV treatment stimulates VASP phosphorylation. (A) Densitometry quantification of protein levels of phosphorylated-VASP (S157) normalized to total VASP of endothelial cells treated for 10 min with vehicle (control), I/R-EVs, or directly with rolipram and isoproterenol to stimulate PKA (I/R-treated). VASP phosphorylation increases with I/R-EV treatment and I/R treatment compared to vehicle *p < 0.05, *n* = 4 (B) Representative western blot. Protein levels in cells treated with vehicle (C), IR (I/R), or I/R-EVs (tEVs); *n* = 4. (C) PKA activity is increased in cell lysates treated with Isoproterenol (Iso) or I/R EVs; *n* = 3, *p < 0.05.

EVs derived from I/R-treated cells were applied to naïve PMVECs for 10 min, the cells were washed, and immunoblotting was performed on lysates to determine the change in VASP protein phosphorylation (Fig. 5A and B). Lysates from cells directly treated with isoproterenol and rolipram were used as a positive control (I/R treated). Levels of phosphorylated VASP in EV-treated cells were significantly increased compared to vehicle-treated control. In fact, VASP phosphorylation in EV-treated cells was remarkably similar to that in the I/R directly treated cells (Fig. 5). These data demonstrate that EV treatment triggers increased phosphorylation of the PKA consensus site.

Next, we sought to confirm that increased phosphorylation of VASP was due to an increase in PKA activity. Following the same treatment as described for the VASP phosphorylation, cell lysates were analyzed with a PKA ELISA. Both isoproterenol and IR-EVs significantly increased PKA activity. Combined these data indicate that I/R-EVs containing cAMP increase cAMP content of naïve cells, and this increase is sufficient to induce PKA-mediated signaling events.

### 3.4. Mechanisms of EV-induced PKA signaling

**Fig. 6.**
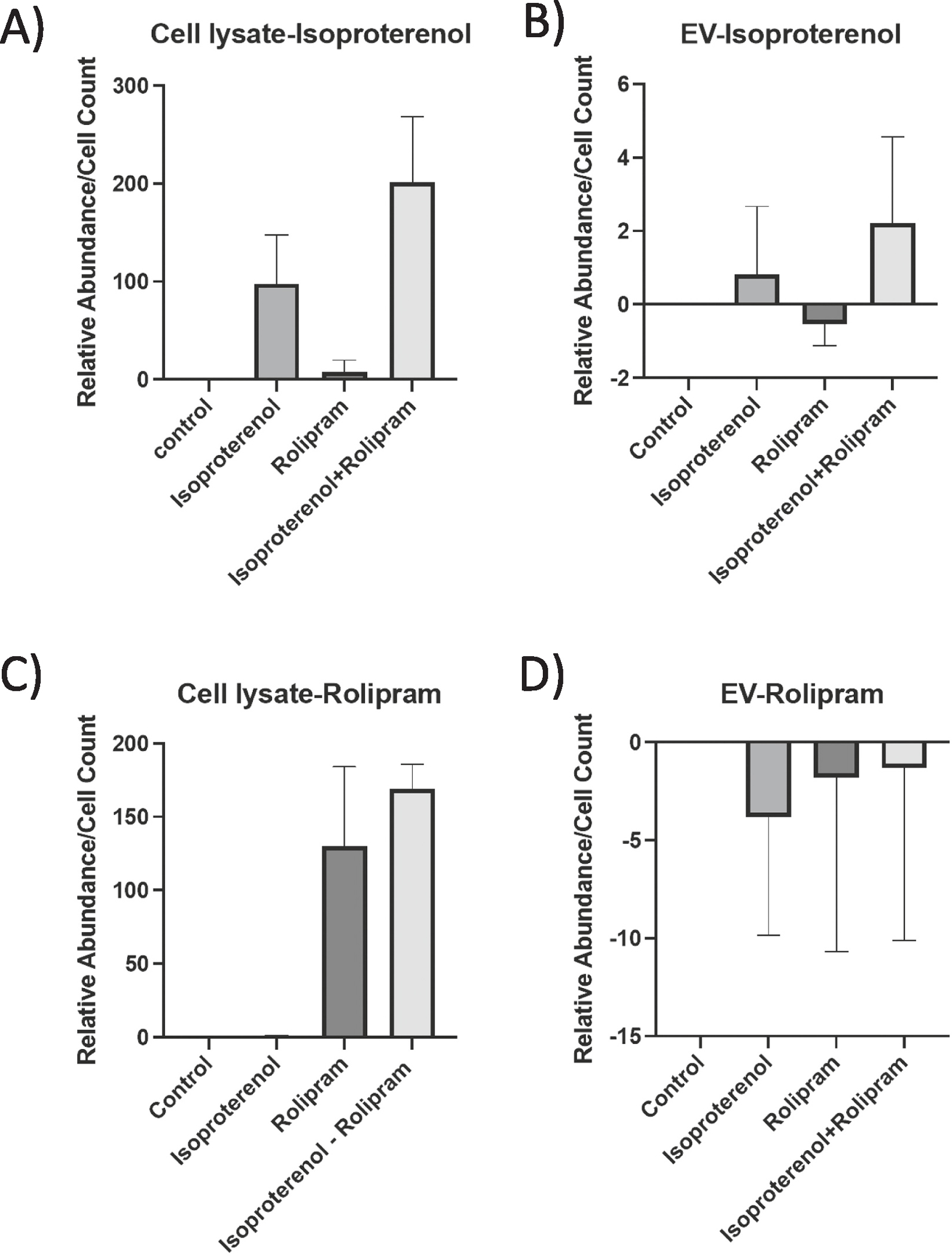
EVs do not deliver I/R. Mass spec analysis of cell lysates indicate both rolipram and isoproterenol are present in cell lysates(A and C), however, isoproterenol is not in abundance in EVs and rolipram is undetectable (B and D). Figs. A and B represent mass spec runs for isoproterenol detection and Figs. C and D for rolipram. Relative abundance is the amount of substance (Iso or Rol)/total cell count used for the experiment.

Since treatment with I/R increases cellular cAMP content, we first considered the possibility that EVs from isoproterenol and rolipram-stimulated cells may simply be delivering I/R to the cells to activate a response. Using mass spec we analyzed control (untreated), isoproterenol only, rolipram only, and combination I/R cell lysates and isolated EVs. We confirmed that while there is detectable isoproterenol or rolipram and I/R in the cell lysates (Fig. 6A and C), the relative abundance of isoproterenol in isolated EVs is insignificant and undetectable at all for rolipram (Fig. 6B and D). These data suggest that EVs are not simply delivering I/R to recipient cells.

**Fig. 7.**
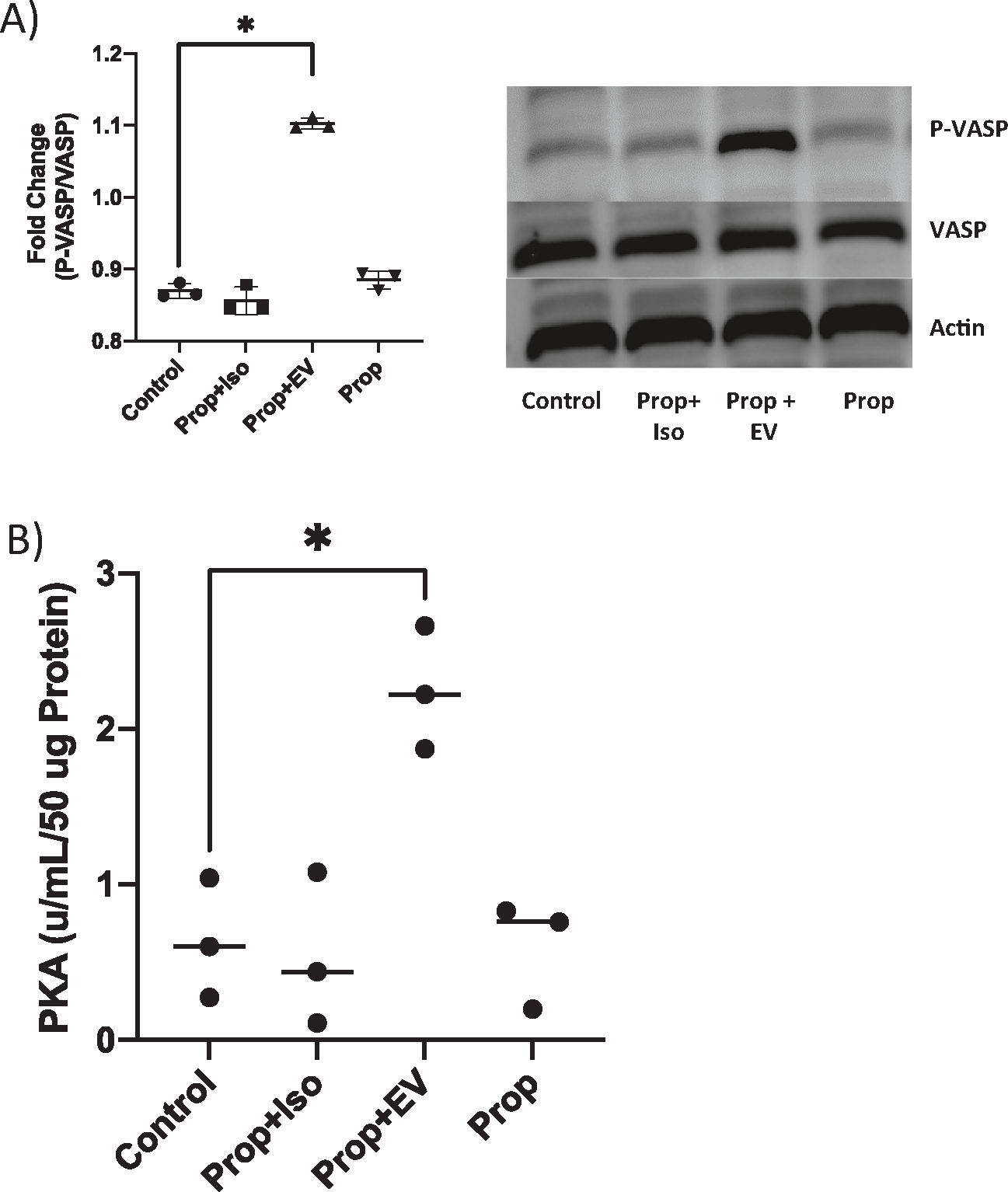
EV-mediated PKA signaling is not β-receptor-dependent. (A) Propranolol, a β-receptor inhibitor was used to pretreat PMVECs (Prop; 25 μM; 1 h) followed by isoproterenol or I/R EVs. Densitometry quantification of protein levels of phosphorylated-VASP (S157) normalized to total VASP and representative Western blot. n = 3 (B) PKA ELISA in the presence of propranolol (Prop; 25 μM; 1 h) reveals that inhibition of the β-receptor does not prevent PKA activity. n = 3, *p *<* 0.05.

Stimulation of adenylyl cyclase is frequently dependent on activation of β-receptors and EVs have the potential to interact with receptors, so we examined this mechanism. We pretreated naïve target cells with propranolol, a nonselective β antagonist [37]. Following pretreatment with the inhibitor, we exposed cells to I/R EVs. Inhibition of β-receptors did not block the downstream phosphorylation of VASP in the presence of EVs but did inhibit p-VASP by direct isoproterenol, a β-agonist, treatment (Fig. 7A). We then analyzed lysates treated in the same manner with the PKA activity assay. Propranolol again inhibited isoproterenol stimulated PKA activity, but did not inhibit IR EV induced PKA. Together, these data suggest that IR EVs can stimulate PKA activity independent of β-receptor activation.

## 4. Discussion

While a multiple proteins and RNA species have been identified in EVs, little attention has been paid to second messenger signaling pathways. Thus, we sought to determine whether cAMP was contained within EVs and, if so, whether this cAMP content was sufficient to induce responses in naïve cells. We previously reported that stimulation of endothelial cells with isoproterenol and rolipram was sufficient to increase the cAMP content of released EVs and that EV numbers did not increase in response to stimulation of parent cells; thus, these data suggest that cells package increased cAMP into released EVs [21]. This may be a passive effect due to the formation of EVs from near membrane areas where compartmentalized regions of increased cAMP exist. However, the packaging of the contents of some EVs is dependent on the stimuli [38]. For example, EVs from endothelial cells stimulated with TNF-α have different membrane markers and 70 uniquely expressed proteins not found in EVs from control cells or those stimulated with plasminogen activator inhibitor-1 [38,39]. These data suggest a more controlled packaging of EV constituents. Future studies evaluating biochemical second messenger packaging are necessary to fully address this concept.

EVs initiate responses in endothelium [14,23,40–44]. However, it was previously unknown whether cAMP contained in EVs was sufficient to stimulate responses in endothelial cells. Thus, we used the cAMP FRET sensor, H188, to measure spatial and temporal changes of cAMP levels in target cells. The cAMP FRET sensor is largely soluble, permitting measurement of cAMP levels throughout the cell [25]. Hyperspectral imaging approaches were used to assess EV-mediated changes in the spatial distributions of FRET signals. Using these approaches, we observed that EVs do indeed trigger marked increases in cAMP levels throughout endothelial cells. Our approach was to pharmacologically maximize the cAMP content of our EVs and determine whether a response was feasible. We also maximized the number of EVs we used per study. Currently, the methodology for accurately separating and diluting EVs into accurate “dose response” studies are fraught with difficulties and methods are under development to address these issues. Future work will not only incorporate novel methodology, but examine physiologic and inflammatory mediators on cAMP-EV generation and endothelial cAMP signaling.

We next sought to determine whether EV-mediated responses were sufficient to trigger changes in the phosphorylation status of consensus PKA phosphorylation sites. While it is intriguing to speculate that EVs can deliver soluble second messenger signals to target cells, thus impacting endothelial function, given the marked difference in the volume of EVs and endothelial cells, it was not clear to us that EVs could contain sufficient cAMP to trigger such responses. Thus, we utilized a mathematical description of cAMP signaling within cells that has been used to describe a variety cellular systems, see [32] and the references therein. Simulations of this model suggest that high levels of cAMP within EVs, 10 to 100 μM, are required to induce significant activation of PKA. While these cAMP concentrations are higher than typical total cellular cAMP levels, several studies have provided evidence that cAMP levels near the plasma membrane can reach levels in the tens of micromolar range [28,30,45,46]. Thus, it is certainly possible that EVs could contain cAMP levels that are high enough to trigger cAMP-mediated responses in target cells. Our models supported the hypothesis the EV-cAMP was sufficient to induce PKA signaling events. Experimentally, we then observed that exposure to EVs resulted in significantly increased phosphorylation of VASP at Ser 157, a consensus PKA site. The level of VASP phosphorylation in EV-exposed endothelial cells was statistically identical to that of VASP phosphorylation achieved by direct stimulation with isoproterenol and rolipram. Our PKA activity assay further confirmed that isoproterenol or cAMP-enriched EVs could induce PKA-mediated signaling. Combined, these data suggest that indeed, EV-cAMP is sufficient to induce PKA signaling events and it seems likely that EV-stimulated PKA-mediated events can subsequently alter cell function and/or gene expression.

It has been suggested that EVs could serve as efficient carriers of pharmaceuticals, and since we are using pharmacological stimulation of the parent cells, we questioned whether the EVs were simply delivering isoproterenol and/or rolipram to the recipient cells. Using mass spec analysis of each analyte we determined that rolipram was undetectable and isoproterenol nearly undetectable in the EV samples. Both analytes were detectable in the cell lysate, however, confirming that the EVs did not simply carry and deliver either drug.

Considering the potential mechanism of cAMP-enriched EV activity, EVs have been shown to interact with membrane receptors. Since transmembrane adenylyl cyclase is traditionally thought to be stimulated by β-adrenergic receptors, we examined whether inhibition of these receptors on endothelium prevented the EV-induced downstream signaling. Our data suggest that EVs do not directly activate β-receptors to induce PKA activity. There are at least three potential models for EV-induced increases in intracellular cAMP: 1) EVs merging with the cellular membrane and releasing cAMP contents; 2) Uptake of EVs by endocytic or other mechanisms and intracellular processing to release cAMP; or 3) the possibility that EVs contain adenylyl cyclase for generation of intracellular cAMP upon engulfment. The present studies do not address these mechanisms directly, but they are all areas of intense study. The future directions of this work will be towards a more complete understanding of the intracellular mechanisms of this novel pathway and the examination of physiologic agonists. While we have yet to determine the exact mechanisms of the increased cAMP or PKA activity, we have clearly identified a novel signaling pathway for EV-induced cAMP signaling.

## Figures and Tables

**Table 1:** Parameters used in one and two compartment models.

Variable	Parameter	Value or initial condition

*AC*_basal.C1_, AC_basal.C2_	Basal adenylyl cyclase activity in C1 and C2	1.25*10^−3^ μM/s 2.5*10^−4^ μM/s
*V*_1_ *and* *V*_2_	Volume of C1 and C2	0.04 pL and 1.96 pL
*k*	cAMP flux coefficient between C1 and C2	1.4 * 10^−16^ L/s
*k*_I_	Inhibition constant of PDE activity	0.1 μM
*[I]*	concentration of PDE inhibitor	0 or 10 μM, as indicated
*km_1_*, *km_2_*	Michaelis constant for PDE activity in C1 and C2	1 μM, 1 μM
*V_max1_*, *V_max2_*	Maximal rate of cAMP hydrolysis in C1 and C2	1 μM/s, 5*10^−3^ μM/s

## References

[1] Burnouf T, Chou ML, Goubran H, Cognasse F, Garraud O, Seghatchian J, An overview of the role of microparticles/microvesicles in blood components: are they clinically beneficial or harmful? Transfus. Apher. Sci. 53 (2015) 137–145. 26596959 10.1016/j.transci.2015.10.010

[2] Burger D, Schock S, Thompson CS, Montezano AC, Hakim AM, Touyz RM, Microparticles: biomarkers and beyond, Clin. Sci. (Lond.) 124 (2013) 423–441. 23249271 10.1042/CS20120309

[3] Hargett LA, Bauer NN, On the origin of microparticles: from “platelet dust” to mediators of intercellular communication, Pulmon. Circul. 3 (2013) 329–340. 10.4103/2045-8932.114760 PMC3757826 24015332

[4] Han J, Yang S, Hao X, Zhang B, Zhang H, Xin C, Hao Y, Extracellular vesicle-derived microRNA-410 from mesenchymal stem cells protects against neonatal hypoxia-ischemia brain damage through an HDAC1-dependent EGR2/Bcl2 Axis, Front. Cell Dev. Biol. 8 (2020), 579236. 33505958 10.3389/fcell.2020.579236 PMC7829500

[5] Lindoso RS, Collino F, Bruno S, Araujo DS, Sant’Anna JF, Tetta C, Provero P, Quesenberry PJ, Vieyra A, Einicker-Lamas M, Camussi G, Extracellular vesicles released from mesenchymal stromal cells modulate miRNA in renal tubular cells and inhibit ATP depletion injury, Stem Cells Dev. 23 (2014) 1809–1819. 24669934 10.1089/scd.2013.0618 PMC4103261

[6] Klinger JR, Pereira M, Del Tatto M, Brodsky AS, Wu KQ, Dooner MS, Borgovan T, Wen S, Goldberg LR, Aliotta JM, Ventetuolo CE, Quesenberry PJ, Liang OD, Mesenchymal stem cell extracellular vesicles reverse Sugen/hypoxia pulmonary hypertension in rats, Am. J. Respir. Cell Mol. Biol. 62 (2020) 577–587. 31721618 10.1165/rcmb.2019-0154OC PMC7193796

[7] Royo F, Thery C, Falcon-Perez JM, Nieuwland R, Witwer KW, Methods for separation and characterization of extracellular vesicles: results of a worldwide survey performed by the ISEV rigor and standardization subcommittee, Cells 9 (2020). 10.3390/cells9091955 PMC7563174 32854228

[8] Cvjetkovic A, Lotvall J, Lasser C, The influence of rotor type and centrifugation time on the yield and purity of extracellular vesicles, J. Extracell Vesicl. 3 (2014). 10.3402/jev.v3.23111 PMC3967015 24678386

[9] Lotvall J, Hill AF, Hochberg F, Buzas EI, Di Vizio D, Gardiner C, Gho YS, Kurochkin IV, Mathivanan S, Quesenberry P, Sahoo S, Tahara H, Wauben MH, Witwer KW, Thery C, Minimal experimental requirements for definition of extracellular vesicles and their functions: a position statement from the International Society for Extracellular Vesicles, J. Extracell Vesicl. 3 (2014) 26913. 10.3402/jev.v3.26913 PMC4275645 25536934

[10] Thery C, Witwer KW, Aikawa E, Alcaraz MJ, Anderson JD, Andriantsitohaina R, Antoniou A, Arab T, Archer F, Atkin-Smith GK, Ayre DC, Bach JM, Bachurski D, Baharvand H, Balaj L, Baldacchino S, Bauer NN, Baxter AA, Bebawy M, Beckham C, Bedina Zavec A, Benmoussa A, Berardi AC, Bergese P, Bielska E, Blenkiron C, Bobis-Wozowicz S, Boilard E, Boireau W, Bongiovanni A, Borras FE, Bosch S, Boulanger CM, Breakefield X, Breglio AM, Brennan MA, Brigstock DR, Brisson A, Broekman ML, Bromberg JF, Bryl-Gorecka P, Buch S, Buck AH, Burger D, Busatto S, Buschmann D, Bussolati B, Buzas EI, Byrd JB, Camussi G, Carter DR, Caruso S, Chamley LW, Chang YT, Chen C, Chen S, Cheng L, Chin AR, Clayton A, Clerici SP, Cocks A, Cocucci E, Coffey RJ, Cordeiro-da-Silva A, Couch Y, Coumans FA, Coyle B, Crescitelli R, Criado MF, D’Souza-Schorey C, Das S, Datta Chaudhuri A, de Candia P, De Santana EF, De Wever O, Del Portillo HA, Demaret T, Deville S, Devitt A, Dhondt B, Di Vizio D, Dieterich LC, Dolo V, Dominguez Rubio AP, Dominici M, Dourado MR, Driedonks TA, Duarte FV, Duncan HM, Eichenberger RM, Ekstrom K, El Andaloussi S, Elie-Caille C, Erdbrugger U, Falcon-Perez JM, Fatima F, Fish JE, Flores-Bellver M, Forsonits A, Frelet-Barrand A, Fricke F, Fuhrmann G, Gabrielsson S, Gamez-Valero A, Gardiner C, Gartner K, Gaudin R, Gho YS, Giebel B, Gilbert C, Gimona M, Giusti I, Goberdhan DC, Gorgens A, Gorski SM, Greening DW, Gross JC, Gualerzi A, Gupta GN, Gustafson D, Handberg A, Haraszti RA, Harrison P, Hegyesi H, Hendrix A, Hill AF, Hochberg FH, Hoffmann KF, Holder B, Holthofer H, Hosseinkhani B, Hu G, Huang Y, Huber V, Hunt S, Ibrahim AG, Ikezu T, Inal JM, Isin M, Ivanova A, Jackson HK, Jacobsen S, Jay SM, Jayachandran M, Jenster G, Jiang L, Johnson SM, Jones JC, Jong A, Jovanovic-Talisman T, Jung S, Kalluri R, Kano SI, Kaur S, Kawamura Y, Keller ET, Khamari D, Khomyakova E, Khvorova A, Kierulf P, Kim KP, Kislinger T, Klingeborn M, Klinke DJ 2nd, Kornek M, Kosanovic MM, Kovacs AF, Kramer-Albers EM, Krasemann S, Krause M, Kurochkin IV, Kusuma GD, Kuypers S, Laitinen S, Langevin SM, Languino LR, Lannigan J, Lasser C, Laurent LC, Lavieu G, Lazaro-Ibanez E, Le Lay S, Lee MS, Lee YXF, Lemos DS, Lenassi M, Leszczynska A, Li IT, Liao K, Libregts SF, Ligeti E, Lim R, Lim SK, Line A, Linnemannstons K, Llorente A, Lombard CA, Lorenowicz MJ, Lorincz AM, Lotvall J, Lovett J, Lowry MC, Loyer X, Lu Q, Lukomska B, Lunavat TR, Maas SL, Malhi H, Marcilla A, Mariani J, Mariscal J, Martens-Uzunova ES, Martin-Jaular L, Martinez MC, Martins VR, Mathieu M, Mathivanan S, Maugeri M, McGinnis LK, McVey MJ, Meckes DG Jr., Meehan KL, Mertens I, Minciacchi VR, Moller A, Moller Jorgensen M, Morales-Kastresana A, Morhayim J, Mullier F, Muraca M, Musante L, Mussack V, Muth DC, Myburgh KH, Najrana T, Nawaz M, Nazarenko I, Nejsum P, Neri C, Neri T, Nieuwland R, Nimrichter L, Nolan JP, Nolte-’t Hoen EN, Noren Hooten N, O’Driscoll L, O’Grady T, O’Loghlen A, Ochiya T, Olivier M, Ortiz A, Ortiz LA, Osteikoetxea X, Ostergaard O, Ostrowski M, Park J, Pegtel DM, Peinado H, Perut F, Pfaffl MW, Phinney DG, Pieters BC, Pink RC, Pisetsky DS, Pogge von Strandmann E, Polakovicova I, Poon IK, Powell BH, Prada I, Pulliam L, Quesenberry P, Radeghieri A, Raffai RL, Raimondo S, Rak J, Ramirez MI, Raposo G, Rayyan MS, Regev-Rudzki N, Ricklefs FL, Robbins PD, Roberts DD, Rodrigues SC, Rohde E, Rome S, Rouschop KM, Rughetti A, Russell AE, Saa P, Sahoo S, Salas-Huenuleo E, Sanchez C, Saugstad JA, Saul MJ, Schiffelers RM, Schneider R, Schoyen TH, Scott A, Shahaj E, Sharma S, Shatnyeva O, Shekari F, Shelke GV, Shetty AK, Shiba K, Siljander PR, Silva AM, Skowronek A, Snyder OL 2nd, Soares RP, Sodar BW, Soekmadji C, Sotillo J, Stahl PD, Stoorvogel W, Stott SL, Strasser EF, Swift S, Tahara H, Tewari M, Timms K, Tiwari S, Tixeira R, Tkach M, Toh WS, Tomasini R, Torrecilhas AC, Tosar JP, Toxavidis V, Urbanelli L, Vader P, van Balkom BW, van der Grein SG, Van Deun J, van Herwijnen MJ, Van Keuren-Jensen K, van Niel G, van Royen ME, van Wijnen AJ, Vasconcelos MH, Vechetti IJ Jr., Veit TD, Vella LJ, Velot E, Verweij FJ, Vestad B, Vinas JL, Visnovitz T, Vukman KV, Wahlgren J, Watson DC, Wauben MH, Weaver A, Webber JP, Weber V, Wehman AM, Weiss DJ, Welsh JA, Wendt S, Wheelock AM, Wiener Z, Witte L, Wolfram J, Xagorari A, Xander P, Xu J, Yan X, Yanez-Mo M, Yin H, Yuana Y, Zappulli V, Zarubova J, Zekas V, Zhang JY, Zhao Z, Zheng L, Zheutlin AR, Zickler AM, Zimmermann P, Zivkovic AM, Zocco D, Zuba-Surma EK, Minimal information for studies of extracellular vesicles 2018 (MISEV2018): a position statement of the International Society for Extracellular Vesicles and update of the MISEV2014 guidelines, J. Extracell Vesicl. (7) (2018) 1535750. 10.1080/20013078.2018.1535750 PMC6322352 30637094

[11] Andreu Z, Yanez-Mo M, Tetraspanins in extracellular vesicle formation and function, Front. Immunol. 5 (2014) 442. 25278937 10.3389/fimmu.2014.00442 PMC4165315

[12] Schafer U, Grunbacher G, Trattnig C, Fasching U, Novak A, Waldispuhl-Geigl J, Leitinger G, Gully C, Patz S, Factors such as regulatory proteins, mRNA and miRNA are transported by stem cell specific microparticles that are released into the cerebprospinal fluid following traumatic brain injury (TBI), J. Stem Cells Regen. Med. 6 (2010) 39–40. 24693061

[13] Cocozza F, Nevo N, Piovesana E, Lahaye X, Buchrieser J, Schwartz O, Manel N, Tkach M, Thery C, Martin-Jaular L, Extracellular vesicles containing ACE2 efficiently prevent infection by SARS-CoV-2 spike protein-containing virus, J. Extracell Vesicl. 10 (2020), e12050. 10.1002/jev2.12050 PMC7769856 33391636

[14] Mack M, Kleinschmidt A, Bruhl H, Klier C, Nelson PJ, Cihak J, Plachy J, Stangassinger M, Erfle V, Schlondorff D, Transfer of the chemokine receptor CCR5 between cells by membrane-derived microparticles: a mechanism for cellular human immunodeficiency virus 1 infection, Nat. Med. 6 (2000) 769–775. 10888925 10.1038/77498

[15] Al-Nedawi K, Meehan B, Micallef J, Lhotak V, May L, Guha A, Rak J, Intercellular transfer of the oncogenic receptor EGFRvIII by microvesicles derived from tumour cells, Nat. Cell Biol. 10 (2008) 619–624. 18425114 10.1038/ncb1725

[16] Umezu T, Ohyashiki K, Kuroda M, Ohyashiki JH, Leukemia cell to endothelial cell communication via exosomal miRNAs, Oncogene 32 (2013) 2747–2755. 22797057 10.1038/onc.2012.295

[17] Loyer X, Potteaux S, Vion AC, Guerin CL, Boulkroun S, Rautou PE, Ramkhelawon B, Esposito B, Dalloz M, Paul JL, Julia P, Maccario J, Boulanger CM, Mallat Z, Tedgui A, Inhibition of microRNA-92a prevents endothelial dysfunction and atherosclerosis in mice, Circ. Res. 114 (2014) 434–443. 24255059 10.1161/CIRCRESAHA.114.302213

[18] Hergenreider E, Heydt S, Treguer K, Boettger T, Horrevoets AJ, Zeiher AM, Scheffer MP, Frangakis AS, Yin X, Mayr M, Braun T, Urbich C, Boon RA, Dimmeler S, Atheroprotective communication between endothelial cells and smooth muscle cells through miRNAs, Nat. Cell Biol. 14 (2012) 249–256. 22327366 10.1038/ncb2441

[19] Treguer K, Heydt S, Hergenreider E, miRNA secreted in vesicles allow atheroprotective communication in vessel wall, Med. Sci. (Paris) 28 (2012) 584–587. 22805133 10.1051/medsci/2012286010

[20] Waldenstrom A, Genneback N, Hellman U, Ronquist G, Cardiomyocyte microvesicles contain DNA/RNA and convey biological messages to target cells, PLoS One 7 (2012), e34653. 22506041 10.1371/journal.pone.0034653 PMC3323564

[21] Sayner SL, Choi CS, Maulucci ME, Ramila KC, Zhou C, Scruggs AK, Yarbrough T, Blair LA, King JA, Seifert R, Kaever V, Bauer NN, Extracellular vesicles: another compartment for the second messenger, cyclic adenosine monophosphate, Am. J. Phys. Lung Cell. Mol. Phys. 316 (2019) L691–L700. 10.1152/ajplung.00282.2018 PMC6483015 30758991

[22] King J, Hamil T, Creighton J, Wu S, Bhat P, McDonald F, Stevens T, Structural and functional characteristics of lung macro- and microvascular endothelial cell phenotypes, Microvasc. Res. 67 (2004) 139–151. 15020205 10.1016/j.mvr.2003.11.006

[23] Blair LA, Haven AK, Bauer NN, Circulating microparticles in severe pulmonary arterial hypertension increase intercellular adhesion molecule-1 expression selectively in pulmonary artery endothelium, Respir. Res. 17 (2016) 133. 27765042 10.1186/s12931-016-0445-1 PMC5073933

[24] Scruggs AK, Cioffi EA, Cioffi DL, King JA, Bauer NN, Lectin-based characterization of vascular cell microparticle Glycocalyx, PLoS One 10 (2015), e0135533. 26274589 10.1371/journal.pone.0135533 PMC4537305

[25] Klarenbeek J, Goedhart J, van Batenburg A, Groenewald D, Jalink K, Fourth-generation epac-based FRET sensors for cAMP feature exceptional brightness, photostability and dynamic range: characterization of dedicated sensors for FLIM, for ratiometry and with high affinity, PLoS One 10 (2015), e0122513. 25875503 10.1371/journal.pone.0122513 PMC4397040

[26] Leavesley SJ, Britain AL, Cichon LK, Nikolaev VO, Rich TC, Assessing FRET using spectral techniques, Cytom. Part A J. Int. Soc. Analyt. Cytol. 83 (2013) 898–912. 10.1002/cyto.a.22340 PMC4374658 23929684

[27] Annamdevula N, Favreau P, Britain A, Nakhmani A, Leavesley S, Rich T, Hyperspectral imaging and image analysis approaches applied to Fret-based measurements of camp signals in pulmonary microvascular endothelial cells, Am. J. Respir. Crit. Care Med. 191 (2015) A2665.

[28] Rich TC, Fagan KA, Tse TE, Schaack J, Cooper DM, Karpen JW, A uniform extracellular stimulus triggers distinct cAMP signals in different compartments of a simple cell, Proc. Natl. Acad. Sci. U. S. A. 98 (2001) 13049–13054. 11606735 10.1073/pnas.221381398 PMC60822

[29] Feinstein WP, Zhu B, Leavesley SJ, Sayner SL, Rich TC, Assessment of cellular mechanisms contributing to cAMP compartmentalization in pulmonary microvascular endothelial cells, Am. J. Physiol. Cell Physiol. 302 (2012) C839–C852. 22116306 10.1152/ajpcell.00361.2011 PMC3311237

[30] Rich TC, Fagan KA, Nakata H, Schaack J, Cooper DM, Karpen JW, Cyclic nucleotide-gated channels colocalize with adenylyl cyclase in regions of restricted cAMP diffusion, J. Gener. Physiol. 116 (2000) 147–161. 10.1085/jgp.116.2.147 PMC2229499 10919863

[31] Stryer L, Intramolecular resonance transfer of energy in proteins, Biochim. Biophys. Acta 35 (1959) 242–244. 13835345 10.1016/0006-3002(59)90355-5

[32] Saucerman JJ, Greenwald EC, Polanowska-Grabowska R, Mechanisms of cyclic AMP compartmentation revealed by computational models, J. Gener. Physiol. 143 (2014) 39–48. 10.1085/jgp.201311044 PMC3874575 24378906

[33] Rich TC, Webb KJ, Leavesley SJ, Can we decipher the information content contained within cyclic nucleotide signals? J. Gener. Physiol. 143 (2014) 17–27. 10.1085/jgp.201311095 PMC3874573 24378904

[34] Benz PM, Blume C, Seifert S, Wilhelm S, Waschke J, Schuh K, Gertler F, Munzel T, Renne T, Differential VASP phosphorylation controls remodeling of the actin cytoskeleton, J. Cell Sci. 122 (2009) 3954–3965. 19825941 10.1242/jcs.044537 PMC2773194

[35] Sartoretto JL, Jin BY, Bauer M, Gertler FB, Liao R, Michel T, Regulation of VASP phosphorylation in cardiac myocytes: differential regulation by cyclic nucleotides and modulation of protein expression in diabetic and hypertrophic heart, Am. J. Physiol. Heart Circ. Physiol. 297 (2009) H1697–H1710. 19734360 10.1152/ajpheart.00595.2009 PMC2781375

[36] Schlegel N, Burger S, Golenhofen N, Walter U, Drenckhahn D, Waschke J, The role of VASP in regulation of cAMP- and Rac 1-mediated endothelial barrier stabilization, Am. J. Physiol. Cell Physiol. 294 (2008) C178–C188. 17989211 10.1152/ajpcell.00273.2007

[37] Lamy S, Lachambre MP, Lord-Dufour S, Beliveau R, Propranolol suppresses angiogenesis in vitro: inhibition of proliferation, migration, and differentiation of endothelial cells, Vasc. Pharmacol. 53 (2010) 200–208. 10.1016/j.vph.2010.08.002 20732454

[38] Peterson DB, Sander T, Kaul S, Wakim BT, Halligan B, Twigger S, Pritchard KA Jr., Oldham KT, Ou JS, Comparative proteomic analysis of PAI-1 and TNF-alpha-derived endothelial microparticles, Proteomics 8 (2008) 2430–2446. 18563738 10.1002/pmic.200701029 PMC4753841

[39] Jimenez JJ, Jy W, Mauro LM, Soderland C, Horstman LL, Ahn YS, Endothelial cells release phenotypically and quantitatively distinct microparticles in activation and apoptosis, Thromb. Res. 109 (2003) 175–180. 12757771 10.1016/s0049-3848(03)00064-1

[40] Mahmoud AM, Wilkinson FL, McCarthy EM, Moreno-Martinez D, Langford-Smith A, Romero M, Duarte J, Alexander MY, Endothelial microparticles prevent lipid-induced endothelial damage via Akt/eNOS signaling and reduced oxidative stress, FASEB J. 31 (2017) 4636–4648. 28687612 10.1096/fj.201601244RR PMC5714503

[41] Brodsky SV, Zhang F, Nasjletti A, Goligorsky MS, Endothelium-derived microparticles impair endothelial function in vitro, Am. J. Physiol. Heart Circ. Physiol. 286 (2004) H1910–H1915. 15072974 10.1152/ajpheart.01172.2003

[42] Mezentsev A, Merks RM, O’Riordan E, Chen J, Mendelev N, Goligorsky MS, Brodsky SV, Endothelial microparticles affect angiogenesis in vitro: role of oxidative stress, Am. J. Physiol. Heart Circ. Physiol. 289 (2005) H1106–H1114. 15879485 10.1152/ajpheart.00265.2005

[43] Fujimoto S, Fujita Y, Kadota T, Araya J, Kuwano K, Intercellular communication by vascular endothelial cell-derived extracellular vesicles and their MicroRNAs in respiratory diseases, Front. Mol. Biosci. 7 (2020), 619697. 33614707 10.3389/fmolb.2020.619697 PMC7890564

[44] Bartel S, Deshane J, Wilkinson T, Gabrielsson S, Extracellular vesicles as mediators of cellular cross talk in the lung microenvironment, Front. Med. (Lausanne) 7 (2020) 326. 32850874 10.3389/fmed.2020.00326 PMC7417309

[45] Saucerman JJ, Brunton LL, Michailova AP, McCulloch AD, Modeling beta-adrenergic control of cardiac myocyte contractility in silico, J. Biol. Chem. 278 (2003) 47997–48003. 12972422 10.1074/jbc.M308362200

[46] Warrier S, Ramamurthy G, Eckert RL, Nikolaev VO, Lohse MJ, Harvey RD, cAMP microdomains and L-type Ca2+ channel regulation in guinea-pig ventricular myocytes, J. Physiol. 580 (2007) 765–776. 17289786 10.1113/jphysiol.2006.124891 PMC2075464

